# Lesion Pattern, Mechanisms, and Long-Term Prognosis in Patients with Monoparetic Stroke: A Comparison with Nonmonoparetic Stroke

**DOI:** 10.1155/2017/9373817

**Published:** 2017-09-12

**Authors:** Seung-Jae Lee, Dong-Geun Lee, Hye-Jin Moon, Tae-Kyeong Lee

**Affiliations:** ^1^Department of Neurology, Soonchunhyang University Bucheon Hospital, Bucheon, Republic of Korea; ^2^Department of Neurology, Sejong General Hospital, Bucheon, Republic of Korea

## Abstract

**Background:**

Monoparetic stroke is rare but could be misdiagnosed as peripheral neuropathy. We investigated the prevalence, lesion pattern, stroke mechanism, and long-term prognosis in patients with monoparetic stroke.

**Methods:**

586 acute ischemic stroke patients (including 31 with monoparesis) were studied. Monoparetic stroke was defined as a motor deficit in either an arm or a leg but without facial weakness or speech disturbance. Median follow-up period was 32.0 months. Kaplan-Meier survival curves, log-rank tests, logistic regressions, and Cox proportional hazards models were used for clinical outcome analyses.

**Results:**

The mean age (313 men and 273 women) was 67.6 years. Among monoparetic patients, most had cortical (80.6%) and multiple (64.5%) lesions. The main stroke mechanisms were cardioembolism (38.7%) and large artery atherosclerosis (29.0%). Precentral gyrus with additional regions was most frequently involved in monoparesis (45.2%). Upper motor neuron signs were found in only 11 patients (35.5%). Compared with the nonmonoparetic group, these patients had better functional outcomes (6-month modified Rankin scale ≤2) and long-term survival but had comparable risks for further vascular events, including stroke recurrences.

**Conclusions:**

Although monoparetic stroke may have a better functional outcome, the risk of the further vascular event seems similar to nonmonoparetic stroke.

## 1. Introduction

Monoparesis as a manifestation of acute ischemic stroke is a rare disease entity. Depending on the study definition for monoparesis, its reported prevalence varies over a broad range from 0.7 to 4.1% [1–5]. Some studies included monoparetic cases with sensory symptoms [1, 2], while others studied only pure motor monoparesis [3–6]. In addition, most of the studies have focused on arm or hand weakness and have excluded leg monoparesis [1–3, 6]. Only a few studies have included leg monoparesis in their case series [4, 5]. Thus, to date, there has been little stroke data involving monoparesis of the arm or leg, irrespective of sensory changes seen in a consecutive stroke cohort. However, the rare symptomatic presentation has its clinical implications among various stroke manifestations in that it has a confusing feature possibly leading to misdiagnosis as a peripheral neuropathy as shown in many case series [7–10]. Moreover, there has been little data about its prognosis including stroke recurrence.

Accordingly, we tried (1) to estimate the prevalence of monoparetic stroke potentially simulating peripheral neuropathy in our stroke cohort, (2) to investigate its lesion pattern, stroke mechanism, and initial neurologic findings, and (3) to discover its characteristic clinical features and prognosis by doing a cross-sectional and longitudinal comparison between patients with monoparetic and nonmonoparetic strokes.

## 2. Methods

### 2.1. Patients

We initially recruited 623 acute ischemic stroke patients who were consecutively admitted to the neurovascular or cardiovascular center of Sejong General Hospital within 7 days after symptom onset (between January 2011 and August 2015). From these patients, 16 patients with prestroke modified Rankin scale (mRS) ≥ 3 were excluded. Of the 607 patients, 31 patients (5.1%) were prospectively classified as having a monoparetic stroke and meticulously examined. Of 576 patients with nonmonoparetic strokes, 15 with incomplete study and 6 with no follow-up data of more than 6 months were excluded from this analysis. The Institutional Review Board of Sejong General Hospital approved this study, and informed consent was obtained from all included patients or their legal guardians to publish clinical details.

All survivors were followed up by outpatient clinic attendance. However, 126 patients were not followed up by our clinic at the time of this study. Of these, 93 patients' condition could be ascertained by contacting patients themselves or their relatives via telephone interview. We scored mRS using a structured interview for accurate grading issued by the Korean Clinical Research Center for Stroke. 33 patients lost to follow-up were censored at their last clinic visit.

We monitored a major vascular event (stroke, acute coronary syndrome, or peripheral artery occlusion) and mortality after index stroke in our cohort. The nature of the vascular event and the cause of death were principally based on medical records from the treating physician at Sejong General Hospital. In the absence of such records, medical information was acquired from treating physicians at other institutions. Uncertain information was excluded from the study.

### 2.2. Definition of Ischemic Stroke and Monoparetic Stroke

An ischemic stroke was defined as a focal neurologic deficit of an abrupt onset lasting > 24 hours with an evidence of new infarct lesions on brain imaging. A monoparetic stroke was defined as the presence of a motor deficit in either an arm (hand) or a leg, irrespective of sensory involvement but without facial weakness or speech disturbances. All the strokes were classified as large artery atherosclerosis (LAD), cardioembolism, lacune, two or more mechanisms, cryptogenic stroke, and other causes, according to the TOAST subtype classification system [11].

### 2.3. Clinical Assessment

The clinical information included age, gender, history of hypertension, diabetes mellitus and hyperlipidemia (defined as a total cholesterol level > 200 mg/dl or a low-density lipoprotein cholesterol > 130 mg/dl at the time of presentation or a history of treatment), current cigarette smoking, a previous history of stroke and ischemic heart disease (defined as a known history or clinical demonstration of myocardial infarction or angina pectoris), atrial fibrillation, valvular heart disease, heavy alcohol consumption (>26 Soju drinks/month; about 20% alcohol), medication use (anthrombotics and statin) for ≥ 3 months at stroke onset, and the National Institutes of Health Stroke Scale (NIHSS) score at admission. All 586 patients underwent routine 12-lead ECG, transthoracic echocardiography, and 24-hour Holter monitoring. A total of 117 patients (20.0%) who could accept an esophageal transducer had transesophageal echocardiography for further investigation of the cardioembolic source (e.g., left atrial thrombus, atrial septal abnormality, or spontaneous echo contrast) requested by an attending physician.

### 2.4. Brain Imaging

All the included patients underwent 1.5-T magnetic resonance imaging (MRI) on admission. The MRI consisted of the diffusion-weighted image, gradient echo image, fluid-attenuated inversion recovery image, three-dimensional time-of-flight (TOF) intracranial MR angiography (MRA), and contrast-enhanced MRA, including extracranial carotid and vertebral arteries. Stenoses of brain vessels on MRA were classified as intracranial or extracranial atherosclerotic stenosis, based on the location of the arterial stenosis. More than 50% signal loss on MRA was considered to be significant to the categorization of a stenosis pattern.

In monoparetic stroke, lesion pattern and location were described as follows: (1) the presence or absence of cortical involvement, (2) multiple lesions (more than two topographically distinct lesions) or single lesion (uninterrupted lesion visible in contiguous territories) [12], and (3) lesion location: (i) the precentral knob area only, (ii) precentral gyrus with additional regions, (iii) parietal lobe only, (iv) medial frontal lobe (supplied from anterior cerebral artery), and (v) subcortical regions.

### 2.5. Data Analysis

Statistical analyses were performed with SPSS software, version 18.0 (SPSS Inc., Chicago, IL). The independent *t*-test or Chi-square test was used to compare the difference between nonmonoparetic and monoparetic stroke groups. Univariate and multivariate logistic regression analyses were performed to confirm a negative association between monoparetic stroke and poor functional outcomes (6-month mRS ≥ 3). Kaplan-Meier survival curves were plotted for death, stroke recurrence, and further major vascular events in each group. Differences in the outcomes were evaluated using the log-rank test. A Cox proportional hazards model was used to perform univariate and multivariate analyses for mortality. Independent variables for logistic regression and the Cox proportional hazards model included monoparetic stroke, age (≥65 years), female gender, hypertension, diabetes mellitus, hyperlipidemia, prior stroke history, ischemic heart disease, atrial fibrillation, current smoking, heavy alcohol use, and brain vessel stenosis. Unadjusted and adjusted odds ratios, hazards ratio, and 95% confidence intervals were obtained.* P *values < 0.05 were considered to be statistically significant.

## 3. Results

The mean age of 586 patients (313 men and 273 women) was 67.6 years (range: 18–97) at admission.  Table 1 shows the comparison of clinical features between monoparetic and nonmonoparetic stroke patients. Ages at admission were similar in the two groups. In addition, there was no significant difference in the frequency of gender, vascular risk factors, antithrombotic use, the presence or absence of brain vessel stenosis, the stenosis pattern, and stroke subtypes between the two groups. However, statin use before the index stroke was more frequent in patients with monoparetic strokes.

In a monoparetic stroke, the most frequent stroke subtype was cardioembolism (38.7%), followed by LAD (29.0%), cryptogenic stroke (12.9%), lacune (9.7%), and two or more mechanisms (9.7%, cardioembolism and LAD); most patients had nonlacunar strokes (90.3%). Initial NIHSS scores were far lower, and 6-month outcomes (mRS) were much better in this group compared with the nonmonoparetic stroke group. Moreover, monoparetic stroke was negatively associated with poor functional outcomes (6-month mRS ≥ 3) in univariate and multivariate logistic regression analyses (Table 2).

**Figure 1:**
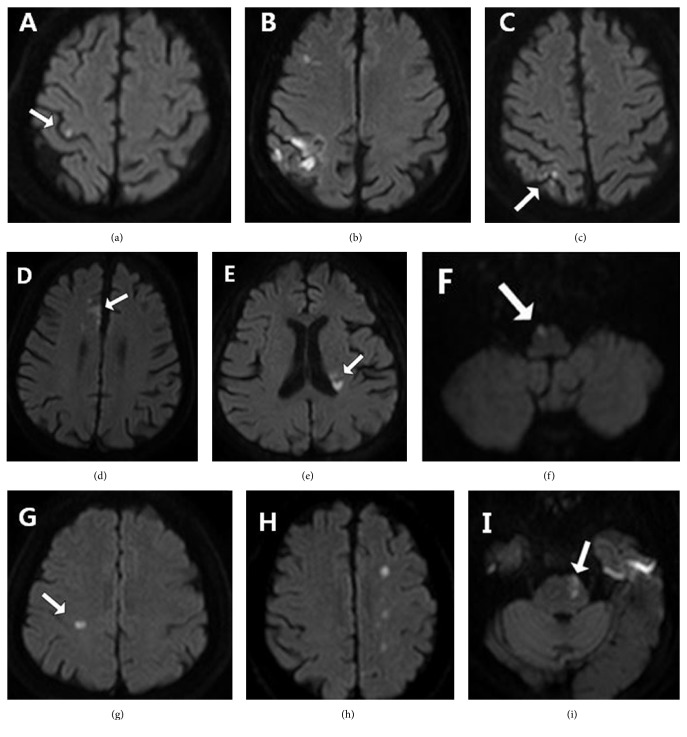
Lesion location and patterns on the diffusion-weighted image in monoparetic stroke. The white arrow indicates acute infarct lesion. (a) Precentral knob area only. (b) Precentral gyrus with additional regions. (c) Parietal lobe only. (d) Medial frontal lobe supplied from the anterior cerebral artery. (e) Corona radiata (posterior medial). (f) Medial medulla. (g) Centrum semiovale. (h) Internal border zone. (i) Rostral anterolateral pons.

Of 31 monoparetic strokes, 19 (61.3%) and 12 (38.7%) involved arm and leg each. 12 patients (38.7%) had no significant proximal weakness in the involved limb (motor score of NIHSS = 0) at admission. Only 3 patients (9.7%) had a grave weakness corresponding to a motor score ≥2 on NIHSS. A total of 10 patients (32.3%) had sensory symptoms. Upper motor neuron signs were found in only 11 patients (35.5%). The most frequently-found upper neuron sign was hyperactive and asymmetric deep tendon reflex (32.3%), followed by the Chaddock sign (12.9%), Hoffman sign (9.7%) and the Babinski sign (3.2%). Most of the patients (80.6%) had a cortical lesion, and 64.5% of the patients had multiple lesions. The most frequently involved region was the precentral gyrus with additional regions (45.2%), followed by the medial frontal lobe (22.6%), subcortical regions (19.4%), the precentral knob area only (9.7%), and the parietal lobe only (3.2%) (Table 3). The precentral knob area involvement was found in 15 patients (48.4%). Subcortical regions were involved in 6 cases. These regions were the posterior medial portion of the corona radiata (*n* = 2), medial medulla (*n* = 1), centrum semiovale (*n* = 1), internal border zone (*n* = 1), and the rostral anterolateral pons (*n* = 1) (Figure 1). Of these, 3 cases (with infarcts in left corona radiata, right medial medulla, and centrum semiovale) were classified as lacune. A total of 12 patients (38.7%) with LAD or a stroke of two or more mechanisms had a relevant atherosclerotic stenosis. The most frequently involved brain vessel was the internal carotid artery (ICA) (7 patients, 22.6%), followed by the anterior cerebral artery (ACA) (4 patients, 12.9%), middle cerebral artery (MCA) (3 patients, 9.7%), and basilar artery (1 patient, 3.2%); two patients had multiple tandem lesions: one had lesions in both ACA and MCA, and the other patient had simultaneous lesions in the ICA, ACA, and MCA.

The median follow-up period was 32.0 months (mean: 32.2; range: 6–70). During that period, 145 of the 555 patients (26.1%) with nonmonoparetic strokes died, whereas only 2 of the 31 patients (6.5%) with monoparetic strokes died due to cardiac disease (large atrial septal defect with Eisenmenger syndrome) and an unknown cause each. The cause of death in nonmonoparetic stroke group was as follows: brain herniation related to index stroke (14 patients, 9.7%), ischemic stroke recurrence (12 patients, 8.3%), heart failure (14 patients, 9.7%), acute myocardial infarction (5 patients, 3.4%), cancer (16 patients, 11.0%), pneumonia (17 patients, 11.7%), sepsis or other infections (7 patients, 4.8%), traumatic intracranial hemorrhage related to a fall (2 patients, 1.4%), spontaneous intracranial hemorrhage (3 patients, 2.1%), hemoptysis (2 patients, 1.4%), other causes (5 patients, 3.4%: aortic dissection in 1, small bowel infarct in 1, traffic accident in 1, renal failure in 1, and suicide in 1), and unknown cause (48 patients, 33.1%).

**Figure 2:**
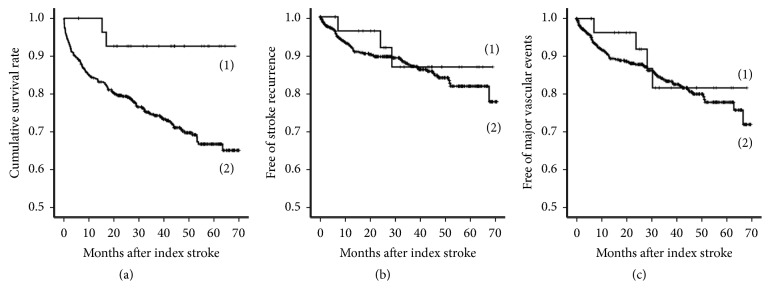
Comparison of Kaplan-Meier curves between monoparetic (1) and nonmonoparetic (2) strokes. *P* value was calculated by log-rank test. (a) Cumulative survival rate: *P* = 0.020. (b) Stroke recurrence: *P* = 0.630. (c) Major vascular events: *P* = 0.657.

Mortality was significantly higher in patients with nonmonoparetic strokes (*P* = 0.020 by log-rank test, Figure 2(a)). The relationship between monoparetic stroke and reduced risk of mortality was proven by using univariate and multivariate Cox proportional hazards models (Table 4). However, the incidences of stroke recurrence and major vascular events were similar between two groups. A total of 65 of the 555 patients (11.7%) with nonmonoparetic strokes and 3 of 31 patients (9.7%) with monoparetic strokes had recurrent strokes. A total of 81 of 555 patients (14.6%) with nonmonoparetic strokes and 4 of 31 patients with monoparetic strokes (12.9%) had a major vascular event (Figures 2(b) and 2(c)).

## 4. Discussion

In our stroke cohort, the prevalence of monoparesis was 5.1%, which is higher than that reported in previous studies [1–5]. This can be attributable to the fact that our study included all cases with a paralysis, not only in an arm but also in a leg (without regard to the presence of a sensory disturbance). As a matter of fact, most of the studies to date included only an arm or a hand paralysis [1–3] and excluded cases with sensory symptoms [3–6, 13]. In addition, not a few studies focused on anatomical location and functional topography of the hand motor area [14–17]. Even so, this rare stroke manifestation is important in real-world clinical practice because of its potential for misdiagnosis as peripheral neuropathy as shown in previous reports [7–10]. Moreover, two-thirds of our monoparetic stroke patients had no upper motor neuron sign. The rate of the absence of upper motor neuron sign in our study was higher compared to a prior study of the prevalence of upper motor neuron sign in patients with acute strokes involving motor functions [18].

In this study, there is no significant difference in basic characteristics between patients with monoparetic and nonmonoparetic strokes, except prestroke statin use. As expected in prior studies, the monoparetic stroke group had a better functional outcome on 6-month mRS compared to nonmonoparetic stroke group [2, 3, 5]. In addition, the main mechanism underlying monoparetic stroke was cardioembolism or LAD, and the main lesion pattern was multiple cortical lesions. Thus, artery-to-artery or cardiac embolism is presumed to be the main cause of monoparetic strokes in our cohort. This finding is consistent with most of the previous results [2, 3, 6, 15], except a study where only selected patients underwent brain MRI and MRA [4]. Therefore, the embolic causes should be determined in comprehensive heart and vessel studies.

Although most of the lesions were located in cerebral cortices, several subcortical regions were also involved. Considering topographical convergence along the descending motor fiber pathway, a cerebral infarct causing monoparesis in the subcortical region tends to be rare and, if any, would possibly be small and solitary. Thus, only six of our patients (19.4%) had a lesion in such regions, and, of these, 3 cases (50%) were classified as lacune. Particularly, a somatotopic organization of motor fibers in corona radiata and rostral pons is relatively well known. Namely, motor fibers for arm and leg are arranged along the anterolateral-to-posteromedial axis in corona radiata and anteromedial-to-dorsolateral axis in the rostral pons, respectively [19, 20]. Consistent with the somatotopy, 2 patients had an infarct in a far posteromedial portion of left corona radiata causing crural monoparesis (Figure 1(e)), and 1 patient had a lesion in left rostral anterolateral pons also leading to crural monoparesis (Figure 1(i)). Besides, monoparesis related to medial medullary infarct has rarely been reported (Figure 1(f)) [21].

Patients with monoparetic strokes had a significantly lower risk of mortality during a mean follow-up period of 2.7 years. Stroke recurrence and major vascular events were observed in 3 (9.7%) and 4 (12.9%) patients with monoparetic strokes, respectively. The recurrence rate is lower than that previously reported in another study (14% of recurrence over 1.7 years mean follow-up) [3]. However, the risks are not lower compared to our patients with nonmonoparetic strokes. This is in line with the findings in our study demonstrating that patients with monoparetic strokes had a similar frequency of vascular risk factors and stenosis in brain vessels compared to patients with nonmonoparetic strokes.

The main limitation of our study was based on a small sample data of a single center, potentially leading to selection bias. Only 31 patients were recruited with monoparetic strokes. Therefore, our results might not be generalizable to other stroke populations. However, most studies to date have consisted of a small number of case series. In addition, in two prior large studies that included more than 50 patients [4, 5], only selected patients underwent MRI and angiographic studies, leading us to conclude that the results did not seem reliable. In contrast, our patients all underwent brain MRIs, intracranial and extracranial MRAs, and full cardiac studies including transthoracic echocardiography and 24-hour Holter monitoring. Moreover, we demonstrated for the first time, through long-term follow-up, that monoparetic stroke, albeit having even better outcomes in mortality and 6-month mRS, was not associated with a lower risk of recurrence or major vascular events compared to nonmonoparetic strokes.

## 5. Conclusion

Acute onset monoparesis should be carefully examined for the probability of stroke, though it is infrequently encountered, because of the paucity of upper motor neuron sign. Since monoparetic stroke seems mostly associated with cardiac or artery-to-artery embolism, detailed studies to detect potential embolic sources should be performed. It could have a better outcome in mortality and functional recovery. However, the risk of further vascular events including stroke recurrence does not seem to be lower compared to nonmonoparetic strokes.

## Figures and Tables

**Table 1:** Comparison of clinical features between monoparetic and nonmonoparetic stroke patients at admission: number (%), mean ± SD.

	Nonmonoparetic stroke *N* = 555	Monoparetic stroke *N* = 31	*P*
Age	67.5 ± 13.7	68.8 ± 11.8	0.611
Female	260 (46.8)	13 (41.9)	0.594
Hypertension	371 (66.8)	23 (74.2)	0.396
Diabetes	164 (29.5)	12 (38.7)	0.279
Hyperlipidemia	287 (51.7)	21 (67.7)	0.082
Current smoking	150 (27.0)	8 (25.8)	0.882
Previous stroke	85 (15.3)	2 (6.5)	0.177
Ischemic heart disease	115 (20.7)	10 (32.3)	0.127
Atrial fibrillation	192 (34.6)	9 (29.0)	0.525
Valvular heat disease	113 (20.4)	5 (16.1)	0.568
Alcohol	92 (16.6)	3 (9.7)	0.310
Previous medication			
Antiplatelet	221 (39.8)	15 (48.4)	0.344
Anticoagulant	80 (14.4)	6 (19.4)	0.449
Statin	145 (26.1)	16 (51.6)	0.002
Stenosis	286 (51.5)	14 (45.2)	0.490
Stenosis pattern			0.620
Intracranial and extracranial	82 (14.8)	3 (9.7)	
Intracranial only	157 (28.3)	7 (22.6)	
Extracranial only	47 (8.5)	4 (12.9)	
No stenosis	269 (48.5)	17 (54.8)	
TOAST classification			0.505
Large artery atherosclerosis	128 (23.1)	9 (29.0)	
Lacune	85 (15.3)	3 (9.7)	
Cardioembolism	207 (37.3)	12 (38.7)	
Two or more	92 (16.6)	3 (9.7)	
Cryptogenic	35 (6.3)	4 (12.9)	
Other causes	8 (1.4)	0 (0)	
Nonlacunar	470 (84.7)	28 (90.3)	0.392
Initial NIHSS	6.6 ± 7.9	1.3 ± 1.0	<0.001
Poor outcome	191 (34.4)	2 (6.5)	0.001

NIHSS: the National Institutes of Health Stroke Scale. Poor outcomes indicate modified Rankin scale ≥3 at 3 months.

**Table 2:** Logistic regression analysis for poor outcomes (6-month mRS ≥ 3).

	Univariate	*P*	Multivariate	*P*
	OR (95% CI)		OR (95% CI)	
Monoparesis	0.132 (0.031–0.561)	0.006	0.123 (0.028–0.543)	0.006
Age ≥65 years	3.624 (2.414–5.441)	<0.001	2.120 (1.336–3.363)	0.001
Female	2.540 (1.782–3.622)	<0.001	1.716 (1.120–2.628)	0.013
Hypertension	1.191 (0.821–1.727)	0.358	1.017 (0.655–1.578)	0.940
Diabetes	1.452 (1.004–2.101)	0.048	1.375 (0.900–2.102)	0.141
Hyperlipidemia	1.067 (0.755–1.508)	0.713	0.858 (0.571–1.290)	0.461
Previous stroke	2.296 (1.446–3.644)	<0.001	1.740 (1.053–2.874)	0.031
Ischemic heart disease	1.313 (0.870–1.982)	0.195	1.150 (0.704–1.881)	0.577
Atrial fibrillation	1.666 (1.165–2.383)	0.005	1.748 (1.137–2.686)	0.011
Smoking	0.347 (0.221–0.545)	<0.001	0.722 (0.412–1.265)	0.255
Alcohol	0.333 (0.186–0.595)	<0.001	0.671 (0.339–1.329)	0.253
Stenosis	2.496 (1.743–3.574)	<0.001	2.322 (1.499–3.597)	<0.001

OR: odds ratio; CI: confidence interval.

**Table 3:** Initial neurologic findings and lesion patterns of patients with monoparetic stroke: number (%).

	Total (*n* = 31)
Location of monoparesis	
Arm	19 (61.3)
Leg	12 (38.7)
Motor score of NIHSS	
0	12 (38.7)
1	16 (51.6)
2	2 (6.5)
3	1 (3.2)
Sensory involvement	10 (32.3)
Any upper motor neuron sign	11 (35.5)
Hyperactive deep tendon reflex	10 (32.3)
Babinski sign	1 (3.2)
Chaddock sign	4 (12.9)
Hoffman sign	3 (9.7)
Lesion pattern	
Cortical involvement	25 (80.6)
Multiple	20 (64.5)
Lesion location	
Precentral knob area only	3 (9.7)
Precentral gyrus with additional regions	14 (45.2)
Parietal lobe only	1 (3.2)
Medial frontal lobe	7 (22.6)
Subcortical regions	6 (19.4)

NIHSS: the National Institutes of Health Stroke Scale.

**Table 4:** Cox proportional hazards models for mortality.

	Univariate	*P*	Multivariate	*P*
	HR (95% CI)		HR (95% CI)	
Monoparesis	0.221 (0.055–0.893)	0.034	0.229 (0.056–0.931)	0.039
Age ≥65 years	3.106 (2.045–4.716)	<0.001	2.143 (1.369–3.355)	0.001
Female	1.911 (1.373–2.661)	<0.001	1.246 (0.867–1.791)	0.234
Hypertension	0.973 (0.693–1.367)	0.875	0.993 (0.681–1.449)	0.971
Diabetes	1.078 (0.760–1.527)	0.674	1.083 (0.751–1.562)	0.669
Hyperlipidemia	0.771 (0.558–1.066)	0.116	0.665 (0.467–0.947)	0.024
Previous stroke	1.492 (0.993–2.240)	0.054	1.174 (0.776–1.777)	0.446
Ischemic heart disease	1.403 (0.970–2.031)	0.072	1.522 (1.012–2.291)	0.044
Atrial fibrillation	1.644 (1.188–2.276)	0.003	1.477 (1.032–2.112)	0.033
Smoking	0.369 (0.230–0.591)	<0.001	0.698 (0.409–1.193)	0.189
Alcohol	0.288 (0.147–0.565)	<0.001	0.467 (0.225–0.968)	0.041
Stenosis	1.629 (1.169–2.268)	0.004	1.449 (0.990–2.120)	0.057

HR: hazard ratio; CI: confidence interval.
